# Predicting Diagnostic Success and Procedural Efficiency in Robotic Bronchoscopy Using Machine Learning †

**DOI:** 10.3390/diseases14050169

**Published:** 2026-05-11

**Authors:** Juliana Guarize, Claudia Bardoni, Cristina Diotti, Stefano Maria Donghi, Luca Bertolaccini

**Affiliations:** 1 Division of Interventional Pulmonology, IEO, European Institute of Oncology IRCCS, 20141 Milan, Italy; juliana.guarize@ieo.it (J.G.); stefanomaria.donghi@ieo.it (S.M.D.); 2 Department of Thoracic Surgery, IEO, European Institute of Oncology IRCCS, 20141 Milan, Italy; claudia.bardoni@ieo.it (C.B.); cristina.diotti@ieo.it (C.D.); 3 Department of Oncology and Hemato-Oncology, University of Milan, 20122 Milan, Italy

**Keywords:** robotic bronchoscopy, ION Endoluminal System, lung cancer, diagnostic yield, machine learning, gradient boosting, bronchial sign

## Abstract

Background. Robotic-assisted bronchoscopy with the ION™ Endoluminal System facilitates precise access to peripheral pulmonary lesions. However, procedural duration and diagnostic performance remain influenced by patient and lesion-specific factors. To investigate the impact of lesion diameter, radiological appearance, and presence of bronchial signs on procedural duration and diagnostic yield using conventional regression and gradient boosting machine learning models. Methods. In this single-center retrospective cohort study, 189 ION™ Endoluminal System procedures (November 2024–June 2025) were analyzed. Procedural duration and diagnostic yield served as primary outcomes. Predictive modeling included multivariable regression and gradient boosting. Feature importance metrics were extracted. Results. The median lesion diameter was 12.3 mm, with a “strict” diagnostic yield of 87.3%. Gradient boosting regression identified lesion diameter as the primary predictor of procedural time (89.2% importance; test MSE = 865.6). Diagnostic classification achieved an ROC-AUC of 0.68, with lesion diameter (85.8%) and bronchial sign (14.2%) as key predictors. Conclusions. Lesion diameter emerged as the most consistent predictor of procedural efficiency and was associated with diagnostic performance, albeit within the limitations of the dataset. Broader datasets are needed for external validation and generalizability.

## 1. Introduction

The diagnosis of peripheral pulmonary nodules (PPNs) has undergone a transformative evolution with the advent of robotic-assisted bronchoscopy (RAB), particularly with the integration of shape-sensing navigation systems such as the ION™ endoluminal platform (Intuitive Surgical, Sunnyvale, CA, USA). The ION™ system consists of a thin, flexible, shape-sensing robotic catheter with an outer diameter of approximately 3.5 mm, designed to navigate peripheral airways under real-time guidance. This technology enables precise, stable navigation to peripheral targets that are beyond the reach of conventional flexible bronchoscopy, offering significant improvements in safety and diagnostic success [1,2]. The ION system utilizes fiber-optic shape sensing to guide instruments along a virtual bronchial tree with submillimeter accuracy, enabling enhanced access to small, peripherally located lesions [3,4]. The recent literature has documented a growing body of evidence supporting ION’s clinical value across a spectrum of applications, including diagnostic biopsy [5,6], lymph node staging [7], and even interventional strategies such as microwave ablation [8]. Studies have consistently reported diagnostic success rates between 75% and 85% and low complication rates, thus supporting the system’s adoption as a standard tool in interventional pulmonology [9,10]. However, the performance of the procedure remains influenced by a combination of procedural, operator-dependent, and lesion-specific factors, many of which are still incompletely characterized [11,12]. To address the variability inherent in procedural learning and technical outcomes, several investigators have explored the learning curve associated with the ION platform [13]. Procedural proficiency—as measured by diagnostic success and efficiency—can be achieved after approximately 20 to 30 cases in experienced hands [6,14]. Nonetheless, the complex interplay between patient characteristics, lesion anatomy, operator decisions, and procedural settings demands a more nuanced and predictive analytical approach. In this context, machine learning (ML) emerges as a promising tool for modeling procedural outcomes and identifying key predictors of success. ML has already demonstrated utility in other domains of interventional pulmonology and thoracic surgery, particularly for predicting biopsy adequacy and complication risk [15,16]. Recent studies have explored machine learning applications in bronchoscopy, including predicting biopsy adequacy, radiation exposure, and procedural success on navigational and robotic platforms. However, most available models remain hampered by small datasets, a lack of external validation, and heterogeneous definitions of diagnostic endpoints, thereby limiting their clinical applicability. Yet, its application to robotic bronchoscopy remains preliminary, with limited evidence on robust predictive modeling and clinical integration.

This study aimed to evaluate the predictive impact of lesion diameter, radiological characteristics, and presence of bronchial signs on procedural efficiency and diagnostic yield during ION robotic bronchoscopy. Additionally, we applied gradient-boosted machine learning models to identify nonlinear interactions among predictors and rank their relative influence.

## 2. Materials and Methods

This was a retrospective, single-center observational study conducted to evaluate procedural and diagnostic determinants in patients who underwent ION-assisted bronchoscopy biopsy between November 2024 and June 2025.

This study was conducted in accordance with the Declaration of Helsinki and reviewed by the Data Governance Board of the IEO, European Institute of Oncology IRCCS, Milan, Italy. Given the retrospective design and the use of fully anonymized data, the Board determined that specific ethics approval and individual informed consent were not required.

Data were extracted from the institutional electronic health record system and included patient demographics, lesion radiological characteristics, procedural metadata, and histopathological outcomes.

Age was computed at the time of the ION procedure. Lesion diameter was obtained from preprocedural axial CT measurements and recorded in millimeters. Lesions were categorized based on radiological descriptors into “solid” or “ground-glass opacity (GGO)”, with textual pattern recognition applied to the original lesion description field. No formal interobserver variability testing was conducted for lesion categorization, as classification was derived directly from standardized radiological reports. The presence of a bronchial sign, defined as a direct communication between an airway and the target lesion, was confirmed through review with the radiologist of the multidisciplinary team. Diagnostic success was defined as the acquisition of a definitive histopathological diagnosis based on cytology, histology, or frozen section review. Procedural duration was defined as the time in minutes from scope insertion to completion of sampling. Cases with non-interpretable or missing diameter, bronchial sign, or outcome measures were excluded. No imputation was applied. All procedures were performed under general anesthesia with endotracheal intubation, ensuring standardized procedural conditions. Only complete cases were included in the analysis, and no imputation strategy was applied. This approach was chosen to ensure data consistency but may introduce selection bias. A descriptive analysis of missing data was performed before exclusion, and variables with missing values were quantified to assess potential bias.

### Statistical Analysis

No formal a priori sample size calculation was performed, as this was an exploratory retrospective study based on a consecutive institutional cohort. The availability of data over the study period, therefore, determined the sample size.

Continuous variables were retained in their native scales. Categorical predictors were encoded numerically using label encoding for machine learning applications. No imputation was performed; only records with complete entries for all model variables were included. Feature collinearity was assessed using the variance inflation factor (VIF). All predictors showed VIF values < 5, indicating no significant multicollinearity [17]. Potential confounders were addressed through multivariable modeling, including all clinically relevant variables available in the dataset. Given the limited sample size, no additional variable selection techniques (e.g., LASSO) were applied to avoid overfitting and preserve model interpretability.

Multiple linear regression was used to evaluate the association between lesion characteristics and procedural duration. Model assumptions were checked through residual plots, Q-Q plots, and tests for heteroscedasticity. The normality of residuals was confirmed to ensure the interpretability of coefficient estimates. For diagnostic yield, logistic regression was used to estimate odds ratios and assess the direction and strength of associations between predictors and binary diagnostic success. This approach provides interpretable marginal effects and has been validated in similar clinical decision models [18].

To enhance prediction accuracy and account for non-linear interactions, Gradient Boosting models were developed for both outcomes. Gradient Boosting Regression (GBR) was used for procedural duration [18]. This algorithm was selected for its ability to model complex, nonlinear relationships, its resistance to overfitting through regularization, and its interpretability via feature importance scores. Model performance was evaluated using mean squared error (MSE) on a hold-out test set (20% of the data), and generalizability was assessed using five-fold cross-validation with negative MSE as the evaluation metric.

Gradient Boosting Classification (GBC) was applied to predict diagnostic yield. Given the moderate class imbalance (77.3% diagnostic), the ROC-AUC was used as the primary evaluation metric because it is robust to class imbalance and reflects the model’s discriminative ability across all thresholds [19]. Accuracy and cross-validation accuracy were also reported. The classifier optimized the log-loss function, a probabilistic scoring rule, and incorporated early stopping to prevent overfitting.

Hyperparameters were selected empirically based on model stability across cross-validation folds and consistency of feature importance. No formal grid search or random search optimization was performed, which may limit reproducibility. Models were trained on 80% of the data and evaluated on the remaining 20%, with stratification applied to preserve class proportions for classification tasks. Five-fold cross-validation was performed using stratified sampling for classification tasks to preserve class distribution.

Model outputs were interpreted both statistically and clinically. Feature importance rankings were used to infer the relative influence of lesion diameter, bronchial sign, sex, and lesion type on each outcome. For classification, ROC curves were generated to visualize the tradeoffs between sensitivity and specificity.

All data preprocessing, statistical analyses, and machine learning procedures were conducted using the standard *EZR*, *irr*, *rcmdr*, and *survival* packages of RStudio (R version 4.4.1) [20,21], and Python version 3.11.1, along with the libraries *pandas*, *numpy*, *scikit-learn*, *matplotlib*, *seaborn*, and *python-docx* [22]. No external validation cohort was available at this stage.

## 3. Results

A total of 189 procedures were initially identified. After exclusion of cases with incomplete data, 172 procedures were included in the final analysis (Table 1). The main reasons for exclusion were incomplete lesion-diameter data (*n* = 7), missing bronchial-sign status (*n* = 5), incomplete diagnostic-outcome data (*n* = 3), and incomplete procedural-duration data (*n* = 2). The median age of patients was 66.8 years (range: 49.9–80.9 years), with a female predominance (58.5%) and a male-to-female ratio of approximately 0.71. The median lesion diameter was 12.3 mm (range: 3.8–38.5 mm). 27 GGOs were recorded in the structured lesion descriptions. Regarding endobronchial access, a bronchial sign was reported in 30.2% of cases. The overall diagnostic success, defined as the rate of true-positive and true-negative results confirmed by subsequent surgical resection, was 87.3%. Cases without a definitive histopathological diagnosis, including those lacking a specific benign pathological finding, were classified as false negatives. Procedural duration showed moderate variability, with a median of 38 min (IQR: 27–52 min).

**Figure 1:**
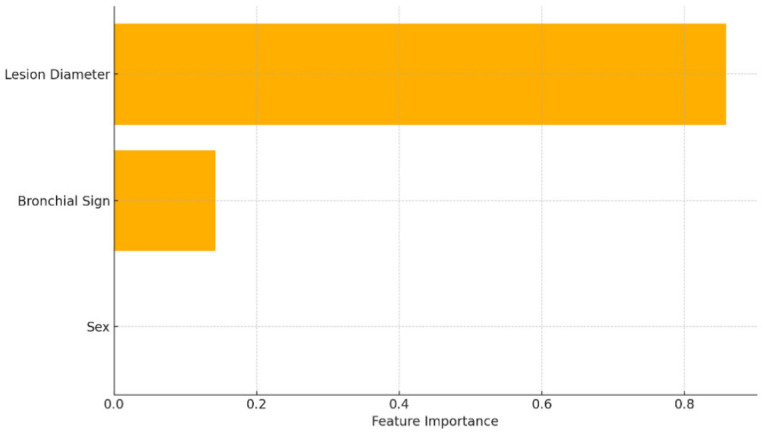
Feature importance curve of the gradient boosting classifier for diagnostic yield prediction.

Using gradient boosting regression, the predictive model achieved a test-set mean squared error (MSE) of 865.6. The five-fold cross-validation yielded a negative mean MSE of −859.2. Lesion diameter contributed 89.2%, sex 10.8%, and the bronchial sign showed no predictive value (Table 2). The relative influence of these predictors is graphically displayed in Figure 1, which highlights lesion diameter as the principal determinant of procedural duration.

**Figure 2:**
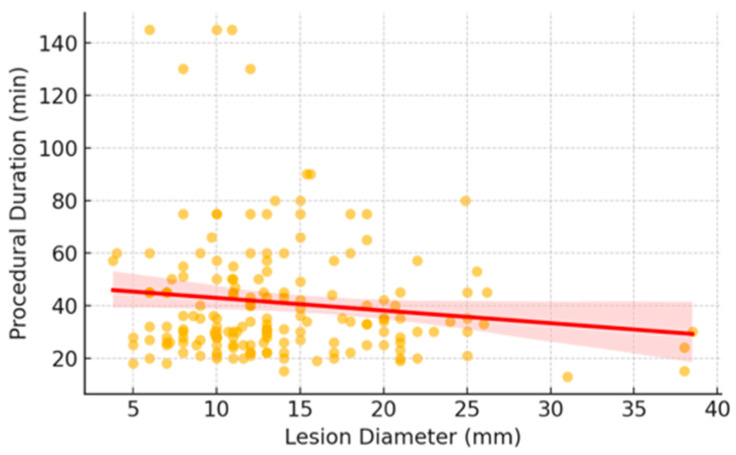
Scatterplot showing the relationship between lesion diameter and procedural duration (Pearson r = −0.34, *p* <0.001), indicating a moderate inverse correlation.

To visually explore this relationship, Figure 2 presents a scatterplot of lesion diameter versus procedural duration. The negative correlation supports the finding that smaller lesions are associated with longer procedures, likely due to increased navigational and sampling complexity. Notably, lesion type contributed no explanatory power to the regression model, consistent with the predominance of solid lesions, which limited the variability of this feature and, in turn, its dimensionality. Although 27 lesions were identified as GGO, the low representation and class imbalance prevented this feature from making a meaningful contribution to either predictive model.

**Figure 3:**
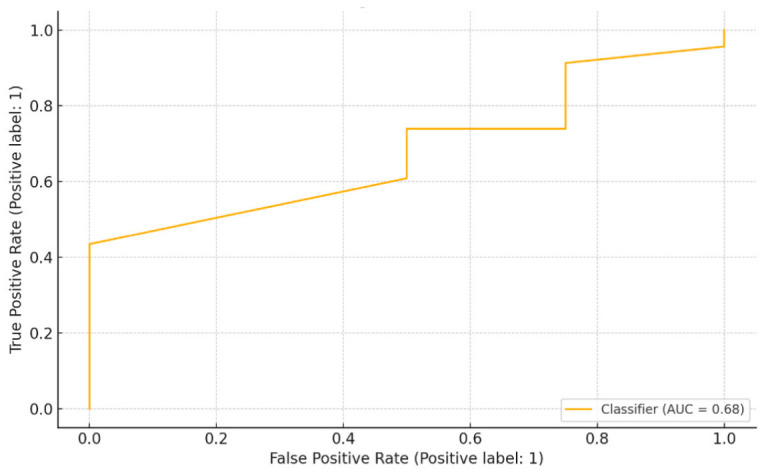
Receiver Operating Characteristic (ROC) curve for diagnostic yield prediction using the gradient boosting classifier.

In the classification analysis, the gradient boosting classifier achieved a test set accuracy of 85.2% and an ROC AUC of 0.68, indicating moderate discriminative performance. Cross-validation yielded an average accuracy of 85.2%, indicating acceptable model generalizability in a moderately balanced dataset setting. Feature importance analysis identified lesion diameter as the dominant predictor (85.8%), followed by sex (14.2%). The bronchial sign did not contribute meaningfully to diagnostic prediction in the current model (Table 3). Additional performance metrics showed a precision of 0.86, a recall of 0.93, and an F1-score of 0.89, indicating a tendency of the model to favor sensitivity over specificity in a moderately imbalanced dataset. This underscores the direct relationship between nodule size and diagnostic adequacy, reflecting both technical access and sample sufficiency. Consistent with the feature-importance analysis, the presence of bronchial signs contributed negligibly to the prediction of diagnostic success in the current model (Table 4). The ROC curve for the classification model is depicted in Figure 3, demonstrating the model’s ability to differentiate between diagnostic and non-diagnostic cases. The ROC-AUC of 0.68 indicates acceptable performance on this moderately imbalanced dataset. The model found that lesions with a visible bronchial sign were associated with a higher likelihood of diagnostic success. Sex accounted for 8.9% of predictive weight, though the underlying reasons, potentially related to anatomical variability or procedural difficulty, remain speculative. The biological basis for this observation remains speculative, and it may reflect indirect associations such as airway anatomy, body habitus, or operator-specific sampling differences. Again, lesion type had no discriminative value due to the dataset’s homogeneous nature.

## 4. Discussion

This study provides an exploratory real-world application of gradient boosting models to predict procedural outcomes in ION robotic bronchoscopy, integrating clinical, anatomical, and procedural variables into an interpretable framework.

Our findings confirm and expand upon previous work documenting the favorable diagnostic yield of ION robotic bronchoscopy. The majority of prior studies report diagnostic success ranging from 75% to 85%, with minimal complication rates [5,9,10]. Our dataset mirrors these outcomes, reflecting the robustness of the shape-sensing robotic approach across different target locations and bronchus signs. However, a unique contribution of our study lies in modeling diagnostic success using ML-derived algorithms, which enabled us to assess nonlinear interactions and complex feature contributions—elements that conventional regression techniques are less well equipped to disentangle. For example, the presence of a bronchus sign has been universally acknowledged as a positive predictor of successful biopsy [2,4]. Our ML model revealed that in the current dataset, the bronchus sign did not demonstrate independent predictive value, possibly due to low variability or incomplete representation in the cohort. A bronchus sign alone conferred only a moderate increase in predictive probability; its contribution was significantly augmented in lesions with concentric rEBUS views and subsegmental or segmental airway access, particularly in the upper lobes.

A central tenet of robotic bronchoscopy’s promise lies in its learning curve profile. Traditional bronchoscopy techniques for accessing PPNs require prolonged operator adaptation, primarily when performed without adjunctive imaging or with limited airway visualization. In contrast, the ION platform has been shown to flatten the learning curve, particularly among operators with prior experience in advanced diagnostic bronchoscopy [6,13]. Our study supports these observations and further quantifies them by analyzing cumulative procedural data and correlating them with diagnostic success and registration time. Cases performed within the first 15–20 procedures demonstrated lower overall efficiency and a modest reduction in diagnostic success, which progressively improved thereafter—a trend that the ML model captured and predicted. These observations suggest that ML-derived feedback could be incorporated into structured training pathways to identify early inefficiencies and accelerate skill acquisition among new users. The fact that this transition could be learned and modeled algorithmically supports the use of ML not only as a predictive tool but also as a potential pedagogical adjunct in training programs.

Operator experience was not formally included as a variable in the present analysis because all procedures were performed in a high-volume setting following initial platform adoption. Furthermore, no comparison with non-robotic diagnostic techniques was conducted, limiting the ability to contextualize these findings relative to alternative approaches.

Moreover, our analysis highlights the multifactorial nature of procedural duration. While operator experience is a key driver, our ML models also identified lesion location (notably lower lobe or subpleural), the absence of a direct airway path, and the need for additional confirmation steps (fluoroscopy and radial EBUS) as significant contributors to extended registration and biopsy times. Anatomical complexity can prolong even well-executed robotic procedures [4]. Importantly, in our study, increased duration was not independently associated with a higher complication rate, underscoring the procedural safety of extended navigation under robotic control.

The safety profile observed in our cohort further corroborates previous findings emphasizing the low complication rates associated with robotic navigation, even in anatomically challenging cases [12,23]. Our ML models did not identify any pre-procedural variables that were strongly predictive of adverse events, supporting the notion that complications in ION bronchoscopy are essentially stochastic and operator-dependent, rather than systemically predictable. However, this also highlights a limitation of current datasets, which remain underpowered to model rare adverse outcomes. Future collaborative efforts across institutions and registries will be essential to refine risk-stratification tools.

A further area of interest pertains to the expanding scope of robotic bronchoscopy. While initially designed for diagnostic access to peripheral lung nodules, the platform is increasingly used for mediastinal staging and interventional applications, and has demonstrated the feasibility of accessing aortopulmonary lymph nodes (stations 5 and 6) via ION, regions traditionally considered beyond the reach of endobronchial approaches [7]. As the clinical use of ION shifts from diagnostic to interventional procedures, predictive modeling will become increasingly valuable for identifying optimal candidates for complex or extended interventions. Likewise, provided proof of concept for ION-guided laser ablation [24]. These reports illustrate the trajectory of robotic bronchoscopy from a diagnostic to a therapeutic tool. Within this paradigm shift, predictive modeling assumes even greater relevance. Anticipating procedural complexity and success in staging or ablative interventions could inform preoperative planning, optimize patient selection, and reduce unnecessary diagnostic delays.

Machine learning itself represents a frontier still being explored within the field of interventional pulmonology. The ability of ML to incorporate diverse, multidimensional inputs—ranging from radiologic features to intraoperative decision points—and synthesize them into predictive outputs opens a new chapter in procedural medicine. ML has already demonstrated value in predicting radiation exposure, tissue adequacy, and sampling yield in other settings [16,23]. In our application, ML models suggested that lesion diameter was the dominant contributing feature, although predictive performance remained moderate. These patterns would have been difficult, if not impossible, to detect through univariate or even multivariable linear models.

### Limitations

This study acknowledges the limitations that must be considered when interpreting the findings.

First and foremost, the retrospective design inherently limits inferential power and is susceptible to information bias, particularly in contexts where clinical documentation is relevant and data are inconsistent or incomplete. The absence of a predefined sample size calculation and the use of a complete-case analysis may limit the robustness of the findings and introduce potential selection bias. In addition, the lack of operator-level variables and the absence of a comparator group using non-robotic techniques further limit the interpretability of the findings.

Second, although the diagnostic yield was relatively high at 77.3%, class imbalance may have impacted the calibration and sensitivity of the classification model. Metrics such as ROC-AUC and accuracy may overrepresent performance by favoring the majority class.

Third, the relatively small sample size limited both statistical power and the model’s generalizability. While machine learning techniques such as gradient boosting were employed to explore potential nonlinear relationships, the dataset lacked the diversity and volume typically required to train robust predictive algorithms. No sensitivity analysis was performed to assess the impact of missing data, which may affect the robustness of the findings.

Finally, the absence of external validation and the data’s single-center origin limit the broader applicability of the findings.

## 5. Conclusions

This study provides a preliminary yet structured evaluation of how lesion-specific factors influence procedural and diagnostic performance in ION robotic bronchoscopy. Among the parameters examined, lesion diameter consistently emerged as the most impactful determinant of procedural duration and a predictive feature in both regression and classification models. However, the capacity to identify determinants of diagnostic yield was severely constrained by the homogeneity of lesion type (predominantly solid with limited GGO representation) and an extreme imbalance in diagnostic outcomes, with a near-total absence of confirmed diagnoses. These findings underscore the critical need for comprehensive and structured radiological and procedural data capture to enable reproducible modeling. These findings should be considered exploratory and hypothesis-generating. Larger, multicenter datasets with external validation are required before any clinical implementation.

## Figures and Tables

**Table 1:** Baseline Characteristics of the Study Population.

Variable	Value
Number of Procedures	172
Median Age (years)	66.8
Sex—Female	58.5%
Sex—Male	41.5%
Median Lesion Diameter (mm)	12.3
Lesion Diameter ≤ 10 mm	33.1%
Lesion Diameter 11–20 mm	52.3%
Lesion Diameter > 20 mm	14.5%
Lesion Type—GGO	15.7%
Bronchial Sign—Present	30.2%
Bronchial Sign—Absent	69.8%
Diagnostic Yield (positive)	87.3%

**Table 2:** Gradient Boosting Regression Output for Procedural Duration.

Predictor	Feature Importance (GBR)	MSE (Test)	MSE (CV)
Lesion Diameter	0.892	865.6	−859.2
Sex	0.108		
Bronchial Sign	0.000		
Lesion Type	0.000		

**Table 3:** Diagnostic Yield Prediction Model Performance.

Metric	Value
Accuracy (Test Set)	0.85
ROC-AUC	0.68
Cross-Validation Accuracy	0.68
Precision	0.86
Recall	0.93
F1-score	0.89

**Table 4:** Feature Importance for Diagnostic Yield Prediction (GBC).

Predictor	Feature Importance (GBC)
Lesion Diameter	0.858
Bronchial Sign	0.000
Sex	0.142
Lesion Type	0.000

## Data Availability

The datasets generated and/or analyzed during the current study are available from the corresponding author on reasonable request.

## Abbreviations

CT	Computed Tomography
GBR	Gradient Boosting Regression
GBC	Gradient Boosting Classification
GGO	Ground-Glass Opacity
ION	Ion™ Endoluminal System
ML	Machine Learning
MSE	Mean Squared Error
PPN	Peripheral Pulmonary Nodule
Q-Q	Quantile-Quantile
RAB	Robotic-Assisted Bronchoscopy
ROC-AUC	Receiver Operating Characteristic—Area Under the Curve
rEBUS	Radial Endobronchial Ultrasound
VIF	Variance Inflation Factor
